# Ameliorative Effect of Crocin on Sperm Parameters and *In Vitro* Fertilization in Mice under Oxidative Stress Induced by Paraquat

**DOI:** 10.22074/ijfs.2020.5617

**Published:** 2019-11-11

**Authors:** Fahime Sadat Kamali, Rasoul Shahrooz, Gholam Reza Najafi, Mazdak Razi

**Affiliations:** 1 Department of Histology and Embryology, Faculty of Veterinary Medicine, Urmia University, Urmia, Iran; 2 Department of Anatomy and Embryology, Faculty of Veterinary Medicine, Urmia University, Urmia, Iran

**Keywords:** Crocin, In vitro Fertilization, Mice, Paraquat, Sperm

## Abstract

**Background:**

Paraquat (PQ) is an herbicide that is genotoxic and cytotoxic for male germ cells. In this study, we investigated the protective role of crocin (Cr) against the destructive effects of PQ on sperm quality and *in vitro* fertilization (IVF) outcomes.

**Materials and Methods:**

In this experimental study, a total of 28 male mice (20-25 g) were divided into four groups: control, which received intraperitoneal (IP) injections of 0.1 ml normal saline per day; PQ group received IP injections of PQ (5 mg/kg/day); experimental (PQ+Cr group) received PQ along with IP injections of Cr (200 mg/kg/day); and positive control (Cr) received IP injections of Cr (200 mg/kg/day). In the last two weeks of the treatment period (35 days of treatment), 16 non-pregnant mice were stimulated to receive adult oocytes. At the end of the treatment period, after euthanizing the mice, the sperms were extracted from the epididymis of each mouse and prepared for evaluation of sperm parameters and IVF.

**Results:**

In the PQ+Cr group, Cr caused a significant increase in the average number of sperms and the mean percent- age of motile and viable sperms. There was a significant decrease in the mean number of immature and DNA-damaged sperms compared to the PQ group (P<0.001). IVF evaluation in the PQ+Cr group showed that the mean percentage of fertilization, two- and four-cell embryos, blastocysts, and hatched embryos significantly increased. Cr caused a significant decrease in the mean percentage of the arrested embryos compared to the PQ group (P<0.001). However, the Cr group did not have any toxic effects on sperm quality or IVF results.

**Conclusion:**

The findings of this study showed that Cr, due to its effective and potent antioxidant properties, could reduce or suppress the destructive effects on sperm parameters and IVF caused by PQ.

## Introduction

Paraquat (PQ) (N, N'-dimethyl-4,4'-bipyridinium dichloride) is a pyridine compound that contains an ammonia sodium moiety, which activates oxidation. Oxidation begins with the methylation of chloromethane. PQ is a nonselective contact herbicide that is used to control annual weeds (1). 

In general, the fertility rate of men exposed to toxins in the workplace is significantly lower (2). PQ is a highly effective, fast-acting, and nonselective herbicide widely used throughout the world. Human exposure by either respiratory or systemic routes leads to the accumulation of PQ in the lungs, resulting in pulmonary oedema, bronchial and alveolar destruction, and ultimately fibrosis with a high mortality, which is in part caused by the lack of a specific antidote to PQ (3). Chronic exposure to PQ is associated with liver damage, kidney failure, and Parkinsonian lesions, in addition to fibrosis (4). Upon entering the cells, PQ undergoes cyclic single-electron reduction/ oxidation through its quaternary ammonium nitrogen atoms and bipyridyl ring, and produces reactive oxygen species (ROS) and PQ radicals. Redox cycling is believed to play an important role in initiating lung damage and fibrosis by paraquat. The mechanism by which oxidative signals from PQ interact with the pathways that underlie the lung fibrogenic response is poorly understood (5). PQ reduces the cellular oxidation cycle with its tangible presence in this process, which results in the production of ROS (6). In a rat model, free radicals have been shown to accumulate in the testicular torsion, causing inflammation and damage to the tissues by membrane lipid peroxidation (7). Because of the scant amount of cytoplasmic antioxidant enzymes, the sperm cannot repair the damage caused by oxidative stress. Studies have shown that antioxidants such as vitamin C have extensive ameliorative effects on sex hormonal status and can protect sperm from ROS-induced abnormalities. These compounds also inhibit ROS produced by leukocytes, improve the quality of semen, and prevent DNA fragmentation and premature sperm production (8). Accordingly, antioxidants such as Fumaria parviflora on testicular injury induced by torsion/ detorsion in adult rats have confirmed reduced DNA damage and apoptosis in sperms, as well as increased implantation and pregnancy (9).

Carotenoids, by acting as biological antioxidants, protect cells and tissues from the damaging effects of free radicals and singlet oxygen, and play a significant role in human health. Crocus sativus L., commonly known as saffron, is a stemless herb of the Iridaceae family. The major bioactive compounds in saffron are crocin, safranal, and picrocrocin. Crocin, glycosyl esters of crocetin, are unusual water-soluble carotenoids, and are responsible for the characteristic colour of saffron (10). Numerous studies have shown that crocin (Cr) can produce a variety of pharmacological effects, such as protection against cardiovascular diseases (11), inhibition of tumour cell proliferation (12), and neuroprotection (13). Saffron has a role in sexual enhancement (14). It also has been reported that Cr inhibits lipid peroxidation in the kidneys (15). The antioxidant and radical scavenging activities of Cr have also been reported in several *in vitro* models (16). In the current study, we aimed to evaluate the protective capacity of Cr on quality and fertilization potential of mice sperm against the toxicity caused by paraquat. 

## Materials and Methods

We purchased PQ with formulation of SL20%, (Exir Co., Iran). Cr was purchased from Sigma-Aldrich (USA) in the form of a powder. 

In this experimental study, 28 adult mice (20 to 25 g) were randomly divided into four equal groups and allowed to adjust to their surroundings for one week before the start date of the experiments. All the ethical issues were carried out based on guidelines of the Ethics Committee of Urmia University, Faculty of Veterinary Medicine (ethics number: ECVU-173-2018). The mice were kept on a 12-hour light/12-hour dark schedule with free access to adequate water and food. The animals were fed with pellets and wheat, and tap water was used as drinking water. The treatment period lasted 35 days. 

The mice were assigned to the following experimental groups: control group, which received intraperitoneal (IP) injections of normal saline (0.1 ml/day); PQ group received PQ (5 mg/ kg/day, IP) (17); experimental (PQ+Cr) group received Cr (200 mg/kg/day, IP) two hours before PQ (5 mg/ kg/day, IP) (18); and positive control (Cr) group received Cr (200 mg/kg/day, IP).

At the end of the treatment all mice were weighed one hour before the beginning of the sampling. They were anesthetized by ketamine (40 mg/kg) and xylazine (5 mg/ kg), and were euthanized by dislocation of their neck vertebrae.

### Sperm preparation

To obtain sperm from the testicles, the abdominal skin was first sterilized with 70% ethanol. After cutting off the surrounding connective tissues, the tail of each epididymis was removed from the testes, and we placed them in sterile test tubes that contained 1 cc of human tubal fluid (HTF) medium (Sigma-Aldrich, USA) with bovine serum albumin (BSA, 4 mg/ml), which had been previously placed in an incubator to equilibrate. The sperm were incubated in a CO_2_ incubator at 37°C. After 30 minutes, the sperm were released and spread in the medium (19).

### Evaluation of sperm parameters

#### Evaluating sperm motility 

In order to evaluate sperm motility, a 10 µl sperm suspension (HTF) and 190 µl distilled water (1:20 dilution) were placed on a pre-heated Neobar slide and covered with a cover slip. Motility was observed under a light microscope (Nikon, Japan) and we counted 10 microscopic fields for each specimen at ×400 magnification (20).

#### Sperm counts

We placed 10 μL of the diluted (1:20) sperm on a neobar slide, after waiting for 5 minutes and counted the number of sperm viewed by an optical microscope at ×400 magnification. We calculated the numbers of sperm according to the following formula: n×50000×d where: n is the number of sperm counted in 5 squares of the Neobar slide and d is the inverse of the dilution of suspension that contained the sperm (in this study d=20).

### Evaluation of sperm viability and morphology

Sperm viability was evaluated as follows. We used eosin-nigrosin staining to detect the nonviable sperm. These sperm are permeable to dye (eosin) because of plasma membrane damage. We dissolved 20 μl of the sperm in 20 μl of the eosin solution on the slide; after 20 to 30 seconds, we added 20 μl of the nigrosin solution. After the appropriate incubation period, we observed sperm viability with a light microscope at ×400 magnification. The nigrosin sperm (n=400) were counted in each sample and the viability percentage was computed (21). For the sperm morphology assessment, we used both the aniline blue and eosin-nigrosin stains. Sperm that appeared abnormal by aniline blue staining were counted and the results were expressed as percentages. With the eosin-nigrosin staining, spermatozoa that contained cytoplasmic debris were counted as immature sperm (22).

### Evaluation of sperm nucleus maturity

Aniline blue staining was used to evaluate the maturity of the sperm nucleus. In spermatogenesis, a basic protein (protamine) is instead of histones of chromatin in the sperm nucleus. Immature sperm have remnants of histone that take up aniline blue stain, which is an important indicator of sperm maturity. Air-dried smears of the sperm samples were fixed with 3% glutaraldehyde in phosphate-buffered saline (PBS) for about 30 minutes. Then, the slides were stained with aniline blue for 5 minutes. The slides were washed with distilled water and examined with a light microscope at ×400 magnification. The percentages of mature sperm (colourless) and immature sperms (blue) were determined (23).

### Assessment of sperm DNA damage

Acridine orange (AO) staining was used to evaluate any break in the double-stranded DNA of the mice sperm. The prepared semen samples were dried, then fixed for 2 hours using a Carnoy’s solution, and subsequently stained with AO for 10 minutes. After the slides were washed with water, we examined them with a fluorescence microscope that had a 460 nm filter. The healthy double-stranded DNA showed a green fluorescent colour, whereas the DNA from single-stranded denatured DNA had a yellow to red colour. The results of the DNA damage were presented in percentages (23).

### Sample preparation steps for *in vitro* fertilization

One hour after sperm capacitation, 6×10^6^ sperm/ml of medium were added to the fertilization drops. After 4 to 6 hours, we observed male and female pronuclei formation (the percentage of zygotes), and, after 24 hours the percentage of two-cell embryos, and within 4-5 days the percentage of blastocysts and hatched embryos were investigated (24). 

### Stimulation of ovulation

After 35 days, the mice were prepared for IVF. After ensuring the setting of the light cycle (12 hours light/12 hours dark) of the female mice that is essential for the regulation of the sexual cycle and lasts for at least 2 weeks. Female mice ovaries were stimulated to obtain mature oocytes. The female mice received injections of 0.2 ml of 10 IU of pregnant mare serum gonadotropin (PMSG, Folligon, The Netherlands) and after 46- 48 hours, they received IP injections of 0.2 ml of 10 IU of human chorionic gonadotropin (hCG, Folligon, The Netherlands). Ovulation occurred 10-12 hours after the hCG injection. The oocytes were removed from the ampullae of the oviducts by dissection, and subsequently transferred to fertilization droplets (HTF medium) (19).

### Ovulation and *in vitro* fertilization

Between 10-12 hours after the hCG injection (the next morning), 7 female mice were anesthetized by injections of ketamine (40 mg/kg) and xylazine (5 mg/kg), and then they were euthanized by displacement of the neck vertebrae. After sterilization of the abdominal area, the uterine tubes were detached (Fig .1A, B) and placed in a 37°C equilibrated medium. Then, the oocytes were dissected from the fallopian tubes, washed, and transferred to fertilization droplets under mineral oil that included HTF medium with 4 mg/ml of BSA (Fig .2). The number of potentially active sperm increased to about 1 million/ml in the medium. Fertilization occurs approximately 4-6 hours after addition of the sperm, and with the observation of two pronuclei. The resultant fertilized oocytes (zygotes) were washed, transferred into fresh medium, and equilibrated (19).

**Fig 1:**
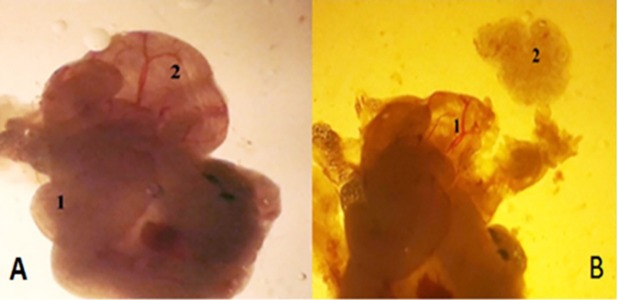
Dissected fallopian tube for obtaining oocyte mass. **A.** Fallopian tubes (1), Ampulla (2) and **B.** Ampulla (1), Detached oocyte masses (2) (magnification: ×40).

**Fig 2:**
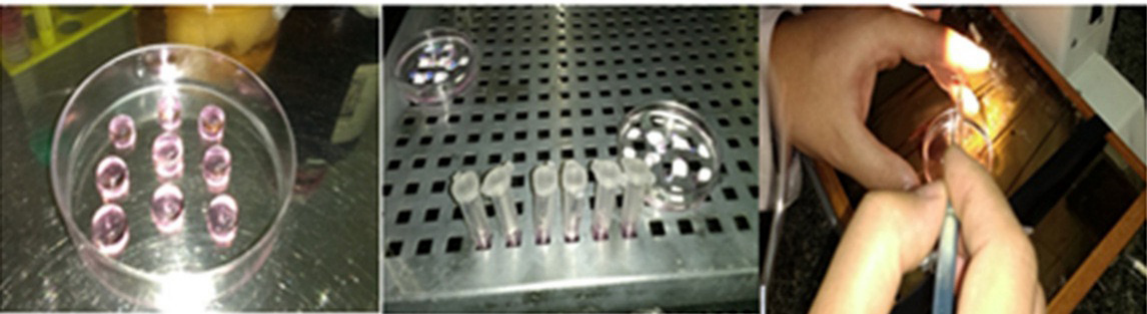
The dissecting steps.

We evaluated the amounts of fragmentation 24 hours after culture, and embryonic development was studied on the fifth day of fertilization. The embryos were examined for the degree of fragmentation, the rate of foetal growth duration or the number, and the type of arrested embryos. We defined the arrested embryos as: type I (embryos with perfect fragmentation and complete necrosis); type II (embryos with fragmentation in several blastomeres); and type III (embryos with a scanty amount of lysis, fragmented blastomeres, and cytoplasmic vesicles).

### Statistical analysis

Data obtained from the sperm evaluation and IVF were analysed by Minitab® software (version 16). All data were compared by nonparametric statistical analysis with the Kruskal-Wallis H test. A P<0.001 was considered significant.

## Results

### Quantitative evaluation of the sperm

The average number of sperm in the PQ group (24.33 ± 1.45%) was significantly different than the control (40.33 ± 2.90%) and PQ+Cr (31.00 ± 1.73%) groups (P<0.001). There was a significant difference between the treatment and the Cr (39.33 ± 1.20%) groups (P<0.001, Table 1). No significant difference existed between the control and Cr groups. 

### Evaluation of sperm motility 

The average percentage of sperm motility showed a significant difference in the PQ (72.33 ± 2.72%) compared with the control (88.00 ± 2.64%) group (P<0.001). The mean percentage of sperm motility was 81.00 ± 1.51% in the treatment group, which was a significant difference compared with the control group (P<0.001). The Cr group (83.66 ± 2.60%) had no significant difference with the control group; however, the treatment group showed a significant difference with the Cr group (P<0.001, Table 1).

### Evaluation of immature sperm

There was a significant difference in average percentage of immature sperm in the PQ group (15.66 ± 0.66%) compared with the control (5.33 ± 0.88%) and PQ+Cr (9.33 ± 0.88%) groups (P<0.001). The Cr group (4.66 ± 0.88%) was not significantly different from the control group (Fig .3A, Table 1).

### Evaluation of sperm viability

The results showed a significant difference in the number of viable sperm between the PQ group (72.00 ± 5.29%) and the control (89.33 ± 2.90%) groups (P<0.001). The mean number of viable sperm showed no significant difference in the treatment group (80.00 ± 2.62%) compared to the control group and no significant difference with the Cr group (88.33 ± 3.52%) as seen in Figure 3B and Table 1.

**Table 1:** Average percentage of data from sperm quality parameters in the different groups


Group	Count×10^6^	Motility	Viability	Immaturity		DNA damage

Con	40.33 ± 2.90 ^ab^	88.00 ± 2.64^ab^	89.33 ± 2.90^a^	5.33 ± 0.88^ab^		2.56 ± 0.46^abc^
25%	35.00	84.00	84.00	4.00		1.8.00
Media	41.00	87.00	90.00	5.00		2.5.00
75%	45.00	93.00	94.00	7.00	3.4.00	
PQ	24.33 ± 1.45^bc^	72.33 ± 2.72^bc ^	72.00 ± 5.29^c^	15.66 ± 0.66^bc^	19.00 ± 2.51^c^	
25%	22.00	67.00	62.00	15.00	16.00	
Media	24.00	74.00	74.00	15.00	17.00	
75%	27.00	76.00	80.00	17.00	24.00	
PQ+Cr	31.00 ± 1.73^c^	81.00 ± 1.15	80.00 ± 2.64	9.33 ± 0.88^c^	13.33 ± 3.38	
25%	28	79.00	75.00	8.00	9.00	
Media	31	81.00	81.00	9.00	11.00	
75%	34	83.00	84.00	11.00	20.00	
Cr	39.33 ± 1.20	83.66 ± 2.60	88.33 ± 3.52	4.66 ± 0.88	6.66 ± 1.20	
25%	37.00	79.00	83.00	3.00	5.00	
Media	40.00	84.00	87.00	5.00	6.00	
75%	41.00	88.00	95.00	6.00	9.00	


Data are presented as mean ± SEM. Con; Control group, PQ; Paraquat group, and Cr; Crocin group. The superscript letters “a, b, and c” indicate a significant difference with the PQ, PQ+Cr, and Cr groups respectively (P<0.001).

**Fig 1:**
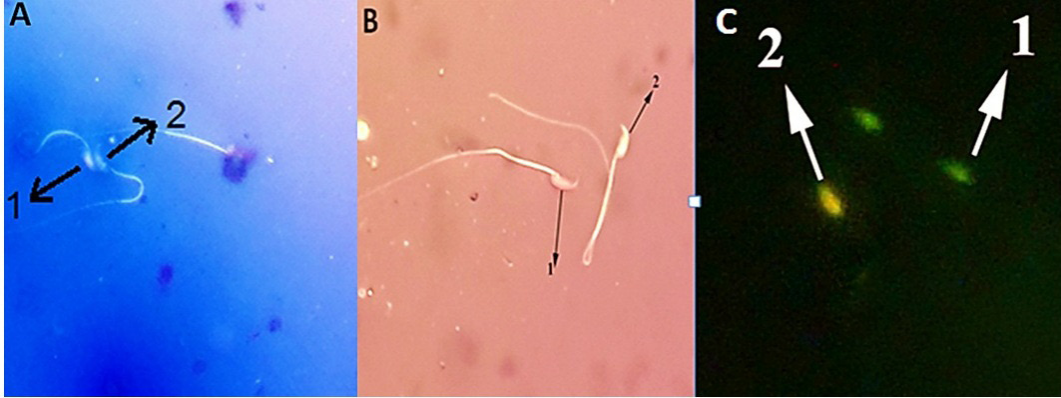
Evaluation of sperm viability, maturity and DNA damage. **A.** Mature sperm are pale (1) immature sperm are light to dark and blue colour (2) (aniline blue, magnification: ×400), **B.** Nonviable sperm are pink to red colour (1) and the viable sperm are achromatic (2) (eosin-nigrosin, magnification: ×400), and C. Sperm without (1) and with (2) DNA damage [Acridine orange (AO), ×400 magnification considering the camera magnification of ×300].

### Evaluation of the number of sperm with damaged DNA

A significant difference in the average number of DNAdamaged sperm was observed in the control group (2.56 ± 1.20%) compared with the PQ (19.0 ± 2.51%), PQ+Cr (13.33 ± 3.38%), and Cr (6.66 ± 1.20%) groups (P<0.001). The PQ group also showed a significant difference compared with the Cr group (P<0.001). The average number of the damaged sperm was not significantly different between the treatment and the Cr groups (Fig .3C, Table 1).

### *In vitro* fertilization

#### Percentage of fertilization

The results of the IVF test showed a significant difference between the PQ group (52.66%) in comparison with the control group (89.87%, P<0.001). The PQ+Cr group showed a significant difference with the PQ group (69.83%, P<0.001). Crocin, alone, did not have a significant effect on fertilization percentage compared to the control group, but it showed a significant difference with the PQ and PQ+Cr (89.48%) groups (P<0.001, Table 2).

#### Percentage of two-cell embryos

**Fig 4:**
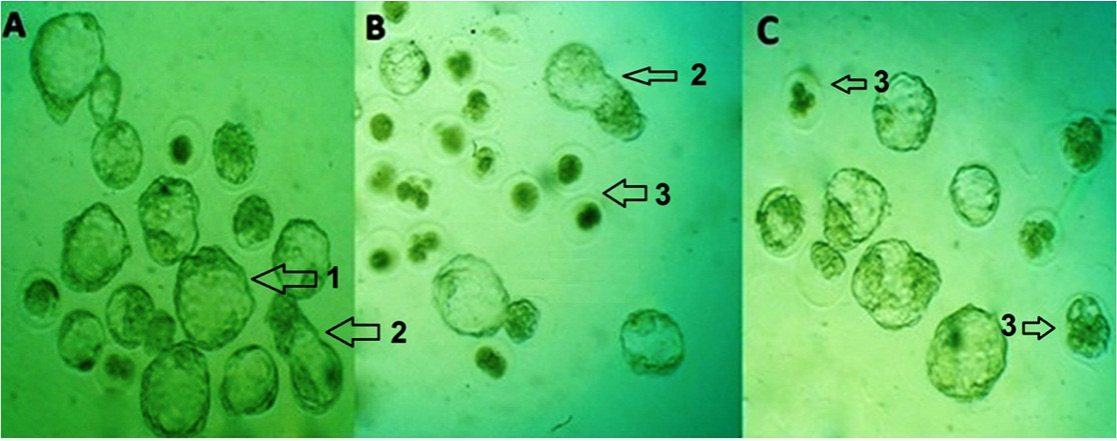
Zygotes and early embryos in different stages of development or arresting. **A.** In the control group, several embryos are seen in the blastocyst and hatching stages, **B.** In the PQ group, several hatched embryos are present at the blastocyst stage, and numerous infertile oocytes and arrested embryos are present and **C.** In the paraquat plus crocin (PQ+Cr) group, several hatched embryos are present in the blastocyst stage. There are several arrested embryos present, (magnification: ×300). 1; Blastocyst, 2; Hatched embryo, and 3; Arrested embryo.

A comparison of the percentage of two-cell embryos that indicated the onset of cleavage showed that the percentage of these embryos were 91.42% in the control group and 77.71% in the PQ group, which was significantly different (P<0.001). The percentage of two-cell embryos in the PQ+Cr group (78.63%) did not show any significant difference with PQ group. There was no significant difference between the Cr (88.77%) and the control group (Fig .4, Table 2).

#### Percentage of four-cell embryos

A comparison of the percentage of four-cell embryos, which indicated the onset of fragmentation revealed that PQ caused a significant difference in the percentage of these embryos, from 82.76% in the control group to 62.60% in the PQ group (P<0.001). Co-administration of Cr and PQ improved the percentage of four-cell embryos to about 63.04% compared to the PQ group, but that difference was not significant. The Cr group (82.99%) showed no significant difference with the control group (Fig .4, Table 2).

#### Blastocyst percentage

The percentage of embryos that reached the blastocyst stage after 120 hours showed a significant difference between the PQ (35.80%) and the control (66.23%) groups (P<0.001). The PQ+Cr group (47.91%) was significantly different compared with the control and Cr groups (P<0.001). The Cr group (71.45%) was significantly different from the PQ and PQ+Cr groups (P<0.001), but the Cr group had no significant difference with the control group (Fig .4A-C, Table 2).

#### Percentage of hatched embryos

PQ caused a significant difference in the percentage of hatched embryos, from 59.78% in the control group to 25.45% in the PQ group (P<0.001). The PQ+Cr group had 39.45% hatched embryos, which differed from the percentages of hatched embryos in comparison with the other groups (P<0.001).The Cr group did not show any significant difference with the control group (59.63%, Fig .4A, B, Table 2).

**Table 2:** Average percentage of obtained data from *in vitro* fertilization (IVF) parameters in the different groups


Groups	Zygote	2-cell	4-cell	Blastocyst	Hatching	Total arrest	Arrest type I	Arrest type II	Arrest type III

Con	89.87 ± 1.60^ab^	91.42 ± 1.98^ab^	82.76 ± 5.11^ab^	66.23 ± 1.39^ab^	59.78 ± 2.05^ab^	35.63 ± 3.45^ab^	15.53 ± 0.62^ab^	20.86 ± 2.56	63.09 ± 2.47^ab^
25%	86.84	87.87	72.72	63.63	57.57	31.15	14.280	16.16	58.33
Median	90.47	91.66	86.11	66.66	57.89	33.33	16.160	21.42	64.28
75%	92.30	94.73	89.47	68.42	63.88	42.42	16.160	25.00	66.66
PQ	52.66 ± 4.95^bc^	77.71 ± 1.61^c^	62.60 ± 2.83^c^	35.80 ± 1.37^bc^	25.45 ± 1.55^bc^	64.18 ± 1.37^bc^	69.80 ± 0.82^bc^	18.51 ± 1.74	11.67 ± 2.41c
25%	46.66	76.00	57.14	33.33	23.80	61.90	68.750	15.38	7.14
Median	48.83	76.19	64.00	36.00	24.00	64.00	69.230	18.75	12.50
75%	62.50	80.95	66.66	38.09	28.57	66.66	71.420	21.42	15.38
PQ+Cr	69.82 ± 0.90^c^	78.63 ± 3.08^c^	63.04 ± 4.55^c^	47.91 ± 2.79^c^	39.45 ± 3.60^c^	52.07 ± 2.79^c^	62.69 ± 2.86^c^	16.18 ± 1.90	18.72 ± 2.69c
25%	68.290	73.33	57.14	44.00	35.71	46.66	57.14	14.28	13.33
Median	69.760	78.57	60.00	46.42	36.00	53.57	62.28	14.28	21.42
75%	71.420	84.00	72.00	53.33	46.66	56.00	66.66	20.00	21.42
Cr	89.48 ± 3.27	88.77 ± 2.27	82.99 ± 1.44	71.45 ± 1.98	59.63 ± 3.76	28.53 ± 1.99	13.38 ± 3.06	20.71 ± 4.01	65.89 ± 5.82
25%	83.87	85.00	80.76	67.50	52.38	26.19	7.69	15.38	57.14
Median	89.36	88.46	82.50	73.07	61.53	26.92	14.28	18.18	63.63
75%	95.23	92.85	85.71	73.80	65.00	32.50	18.18	28.57	76.92


Data are presented as mean + SEM. 2-cell; Two-cell embryo, 4-cell; Four-cell embryo, Con; Control group, PQ; Paraquat group, PQ+Cr; Paraquat and crocin group, and Cr; Crocin group. The letters “^a, b^, and ^c^” in a column indicate a significant difference with the PQ, PQ+Cr, and Cr groups, respectively (P<0.001).

### Percentage of total arrested embryos

The total number of arrested embryos showed a significant difference between the control (35.63%) and the PQ (64.18%) groups (P<0.001). The PQ+Cr group (52.07%) had a significantly different percentage of arrested embryos compared to the PQ group (P<0.001). However, in the Cr group there was no significant difference with the control group (Table 2). 

### Percentage of type I arrested embryos

A comparison of the type I arrested embryos showed that this parameter was significantly different in the PQ group (69.80%) compared with the other groups (P<0.001). There was no significant difference between the control group (15.53%) and the Cr groups (13.38%), but there was a significant difference between the PQ+Cr (62.69%) and the control and Cr groups (P<0.001, Table 2).

### Percentage of types II and III arrested embryos

The percentage of type III arrested embryos in the PQ group had a significant difference with the control and Cr groups (P<0.001). There was no significant difference between the groups for type II arrested embryos. However, there was no significant difference in the percentage of type III arrested embryos between the PQ and the PQ+Cr groups (Table 2).

## Discussion

In the present study, we evaluated the experimental groups in two sections: spermatogenesis and early embryonic growth. The results of the first part of the experiment showed that PQ could significantly decrease sperm quality; thereby, in the second part, the PQ group had decreased percentages of fertilization, two-cell embryos, four-cell embryos, blastocysts, and hatched embryos. This group also had increased percentages of whole arrested embryos, and types I, II, and III arrested embryos. In this study, PQ could significantly reduce the number of sperm, the average percentages of sperm motility, and sperm viability, and could significantly increase the percentages of immature sperm and those with damaged DNA, which significantly differed from the control and Cr groups. The results of the two sections of the experiments showed that Cr in the experimental group significantly improved the damage induced by paraquat. In most of the studied parameters, we observed a significant difference between the PQ+Cr and PQ groups.

In previous studies, it has been reported that oxidative stress in animals can cause infertility by affecting the genital organs (25). In a study conducted on men, it has been shown that psychological stress can reduce both motility and sperm quality, and lead to infertility (26). Genital damage is one of the known side effects of PQ poisoning, which has a toxic effect on reproductive systems of both sexes, and it can disrupt the process of spermatogenesis and oogenesis (27). The effects of low doses of PQ on the spermatozoids of Sprague-Dawley rats were studied. The researchers noted that the mean number of sperm decreased on the seventh and fourteenth days after the injection, and abnormal sperm significantly increased. Sperm mortality also increased with higher doses. In this study, it was found that PQ has genotoxic and cytotoxic effects on male germinal cells (28).

The first indication of an increase in ROS is the loss of sperm motility (29). The production of free radicals in mitochondria damage the DNA of mitochondria, and it can also damage the mitochondrial region of the middle part of the spermatozoid (30). Mitochondrial damage of the middle part of the sperm leads to a progressive decrease in sperm motility in terms of decreasing the numbers of motile sperm and decreasing the vehemence of motility (31). 

Antioxidants in semen are categorized within the endogen antioxidant group. Several studies have shown that antioxidants do not reduce sperm motility; however, they increase Sperm capability (32). Laboratory studies have also confirmed the role of antioxidants in reducing ROS production and improving the evolutionary ability of the foetus (33). Results of a study revealed that administration of citrus flavonoid extract significantly increased the total antioxidant capacity (TAC) and superoxide dismutase (SOD) levels, and sperm percentage, viability, and motility, and decreased MDA levels. This suggested that citrus, as an antioxidant, may be promising for enhancement of healthy sperm parameters (34). 

In the present study, the number of fertilized oocytes in the PQ group significantly differed from the control and Cr groups, which indicated that PQ had a negative effect on fertility. This might be due to an increase of free radicals in the testicular tissue and semen, and ultimately damage the membrane of the gametes; it can reduce the percentage of IVF. However, Cr with PQ compensates for this failure. 

Cr appears to have an antioxidant activity when tested *in vitro*. This activity is linked to half of its sugar content (35). In one study, the antioxidant activity of Cr was evaluated and showed that administration of Cr reduced the level of MDA and increased ferric reduction antioxidant power (FRAP) following stress from renal ischemic reperfusion (36). In one study, the results of IVF showed an increase in fertilization, two-cell embryos, and blastocysts in the group that received Cr and cyclophosphamide (CP) in comparison with the CP-only group. It was also shown that in mice that received Cr along with CP the overall numbers of the arresting embryos was decreased in comparison with the CP-alone group. In this study, administration of Cr along with CP reduced MDA malondialdehyde and sperm with damaged DNA in comparison with the CP group (19). 

In the present study, the average number of blastocytes and embryos at the hatching stage in the PQ group significantly differed from the control group. In both cases, the combined use of PQ with Cr ameliorated this effect. This finding showed the antioxidant effect of crocin, while the use of Cr alone did not show any negative effect on any of the above parameters. The PQ group had a significantly different number of arrested embryos compared to the control group, and this effect might be due to the destructive effect of PQ on the membrane and genome of the embryos, whereas Cr has a recovery effect. PQ also caused a negative effect on the percentage of the type I arrested embryos, and Cr ameliorated the percentage of the type III arrested embryos, which was due to its antioxidant properties.

The onset of fragmentation was also caused by the effect of PQ on the fertilized oocytes, which might be due to the effects of PQ on genetic and intracellular factors. This decrease was also observed in the four-cell zygotes, while Cr showed a better protective effect on two-cell zygotes. The effect of PQ on the cellular stage was such that Cr could not compensate for it at this stage. The two factors that protect the sperm DNA against oxidation are the density of the DNA nucleus and presence of antioxidant agents in semen plasma (37). In a study on diabetic men, it was found that the mechanisms that led to the damage of sperm DNA with increasing ROS, which could be a factor in the glycosylation of the final products of advanced glycation end products (38). In addition, the increase of oxidative stress and high fragmentation of DNA continuously occurs with apoptosis (39).

## Conclusion

According to the findings of this study, we concluded that crocin, as an antioxidant, protected the male genital organs against the impacts of oxidative stress induced by PQ and significantly ameliorated both sperm quality and IVF outcomes in paraquat-treated mice. However, this study should be performed at the serological and molecular levels because there is not an adequate knowledge about the effects of PQ poisoning on *in vivo* embryo development. 

## References

[1] Schenker MB, Stoecklin M, Lee K, Lupercio R, Zeballos RJ, Enright P (2004). Pulmonary function and exercise-associated changes with chronic low-level paraquat exposure. Am J Respir Crit Care Med.

[2] Anderson D, McGregor DB, Purchase IF (1976). Dominant lethal studies with paraquat and diquat in male CD-1 mice. Mutat Res.

[3] He X, Wang L, Szklarz G, Bi Y, Ma Q (2012). Resveratrol inhibits paraquat-induced oxidative stress and fibrogenic response by activating the nuclear factor erythroid 2-related factor 2 pathway. J Pharmacol Exp Ther.

[4] Tanner CM, Kamel F, Ross GW, Hoppin JA, Goldman SM, Korell M (2011). Rotenone, paraquat, and parkinson’s disease. Environ Health Perspect.

[5] He X, Wang L, Szklarz G, Bi Y, Ma Q (2012). Resveratrol inhibits Paraquat-induced oxidative stress and fibrogenic response by activating the nuclear factor erythroid 2-related factor 2 pathway. J Pharmacol Exp Ther.

[6] Bus JS, Gibson JE (1984). Paraquat: model for oxidant-initiated toxicity. Environ Health Perspect.

[7] Moghimian M, Abtahi-Evari SH, Shokoohi M, Amiri M, Soltani M (2017). Effect of Syzygium aromaticum (clove) extract on seminiferous tubules and oxidative stress after testicular torsion in adult rats. Physiol Pharmacol.

[8] Moghimian M, Soltani M, Abtahi H, Shokoohi M (2017). Effect of vitamin C on tissue damage and oxidative stress following tunica vaginalis flap coverage after testicular torsion. J Pediatr Surg.

[9] Shokoohi M, Shoorei H, Soltani M, Abtahi-Eivari SH, Salimnejad R, Moghimian M (2018). Protective effects of the hydroalcoholic extract of Fumaria parviflora on testicular injury induced by torsion/detorsion in adult rats. Andrologia.

[10] Rios JL, Recio MC, Giner RM, Manez S (1996). An update review of saffron and its active constituents. Phytother Res.

[11] Shen XC, Qian ZY (2006). Effects of crocetin on antioxidant enzymatic activities in cardiac hypertrophy induced by norepinephrine in rats. Pharmazie.

[12] Magesh V, Singh JP, Selvendiran K, Ekambaram G, Sakthisekaran D (2006). Antitumour activity of crocetin in accordance to tumor incidence, antioxidant status, drug metabolizing enzymes and histopathological studies. Mol Cell Biochem.

[13] Ahmad AS, Ansari MA, Ahmad M, Saleem S, Yousuf S, Hoda MN, Islam F (2005). Neuroprotection by crocetin in a hemi-parkinsonian rat model. Pharmacol Biochem Behav.

[14] Maleki-saghooni N, Mirzaeii K, Hosseinzadeh H, Sadeghi R, Irani M (2018). A systematic review and meta-analysis of clinical trials on saffron (Crocus sativus) effectiveness and safety on erectile dysfunction and semen parameters. Avicenna J Phytomed.

[15] Hosseinzadeh H, Sadeghnia HR, Ziaee T, Danaee A (2005). Protective effect of aqueous saffron extract (Crocus sativus L.) and Crocin, its active constituent, on renal ischemia-reperfusion-induced oxidative damage in rats. J Pharm Pharm Sci.

[16] Mousavi SH, Tayarani NZ, Parsaee H (2010). Protective effect of saffron extract and Crocin on reactive oxygen species-mediated high glucose-induced toxicity in PC12 cells. Cell Mol Neurobiol.

[17] Ranjbar A (2014). Evidence of oxidative damage in paraquat toxicity. Zahedan Journal of Research in Medical Sciences.

[18] Hosseinzadeh H, Abootorabi A, Sadeghnia HR (2008). Protective effect of Crocus sativus stigma extract and Crocin (trans-Crocin 4) on methyl methanesulfonate-induced DNA damage in mice organs. DNA Cell Biol.

[19] Bakhtiary Z, Shahrooz R, Ahmadi A, Soltanalinejad F (2014). Protective effect of Crocin on DNA damage of sperm and in vitro fertilization (IVF) in adult male mice treated with cyclophosphamide. Journal of Mazandaran University of Medical Sciences (JMUMS).

[20] World Health Organisation (1999). WHO laboratory manual for the examination of human semen and sperm-cervical mucus interaction.Cambridge: Cambridge University Press.

[21] Wyrobek AJ, Gordon LA, Burkhart JG, Francis MW, Kapp Jr RW, Letz G (1983). An evaluation of the mouse sperm morphology test and other sperm tests in nonhuman mammals: a report of the U.S.Environmental Protection Agency Gene-Tox Program. Mutat Res.

[22] de Rooij DG, Russell LD (2000). All you wanted to know about spermatogonia but were afraid to ask. J Androl.

[23] Sadeghi MR, Hodjat M, Lakpour N, Arefi S, Amirjannati N, Modarresi T, Jadda HH, Akhondi MM (2009). Effects of sperm chromatin integrity on fertilization rate and embryo quality following intracytoplasmic sperm injection. Avicenna J Med Biotechnol.

[24] Hedrich H, Bullock G (2004). The laboratory mouse: Handbook of experimental animals.

[25] Ma Q, Shao H, Feng Y, Zhang L, Li P, Hu X (2018). A new bioluminescent imaging technology for studying oxidative stress in the testis and its impacts on fertility. Free Radic Biol Med.

[26] Pook M, Tuschen-Caffier B, Krause W (2004). Is infertility a risk factor for impaired male fertility?. Hum Reprod.

[27] Bus JS, Cagen SZ, Olgaard M, Gibson JE (1976). A mechanism of paraquat toxicity in mice and rats. Toxicol Appl Pharmacol.

[28] D’Souza UJ, Narayana K, Zain A, Raju S, Nizam HM, Noriah O (2006). Dermal exposure to the herbicide-Paraquat results in genotoxic and cytotoxic damage to germ cells in the male rat. Folia Morphol (Warsz).

[29] Saleh RA, Agarwal A (2002). Oxidative stress and male infertility: from research bench to clinical practice. J Androl.

[30] Rajesh Kumar T, Doreswamy K, Shrilatha B, Muralidhara A (2002). Oxidative stress associated DNA damage in testis of mice: induction of abnormal sperms and effects on fertility. Mutat Res.

[31] Koppers AJ, De Iuliis GN, Finnie JM, McLaughlin EA, Aitken RJ (2008). Significance of mitochondrial reactive oxygen species in the generation of oxidative stress in spermatozoa. J Clin Endocrinol Metab.

[32] Lenzi A, Sgrò P, Salacone P, Paoli D, Gilio B, Lombardo F (2004). A placebo-controlled double-blind randomized trial of the use of combined l-carnitine and l-acetyl-carnitine treatment in men with asthenozoospermia. Fertil Steril.

[33] SIKKA SC, Rajasekaran MA, Hellstrom WJ (1995). Role of oxidative stress and antioxidants in male infertility. J Androl.

[34] Khaki A, Fathiazad F, Ouladsahebmadarek E, Nouri M, khaki AA, ghanbari Z (2011). Anti-oxidative effects of citro flavonoids on spermatogenesis in rat. Afr J Pharm Pharmacol.

[35] Chen Y, Zhang H, Tian X, Zhao C, Cai L, Liu Y (2008). Antioxidant potential of Crocins and ethanol extracts of Gardenia jasminoides ELLIS and Crocus sativus L.: A relationship investigation between antioxidant activity and Crocin contents. Food Chem.

[36] Najafi H, Yarijani ZM, Najafi M (2017). Theoretical and experimental in vivo study of antioxidant activity of crocin in order to propose novel derivatives with higher antioxidant activity and their delivery via nanotubes and nanocones. Inflammation.

[37] Twigg JP, Irvine DS, Aitken RJ (1998). Oxidative damage to DNA in human spermatozoa does not preclude pronucleus formation at intracytoplasmic sperm injection. Hum Reprod.

[38] Mallidis C, Agbaje I, Rogers D, Glenn J, McCullough S, Atkinson AB (2007). Distribution of the receptor for advanced glycation end products in the human male reproductive tract: prevalence in men with diabetes mellitus. Hum Reprod.

[39] Mallidis C, Agbaje IM, Rogers DA, Glenn JV, Pringle R, Atkinson AB, Steger K, Stitt AW, McClure N (2009). Advanced glycation end products accumulate in the reproductive tract of men with diabetes. Int J Androl.

